# Same-day discharge after atrial fibrillation ablation under a nurse-coordinated standardized protocol

**DOI:** 10.1093/europace/euae083

**Published:** 2024-04-04

**Authors:** Teresa Espinosa, Anna Farrus, Montserrat Venturas, Alba Cano, Sara Vazquez-Calvo, Margarida Pujol-Lopez, Frida Eulogio-Valenzuela, Jean-Baptiste Guichard, Pasquale V Falzone, Freddy R Graterol, Xavier Freixa, Jose M Tolosana, Eduard Guasch, Andreu Porta-Sanchez, Elena Arbelo, Josep Brugada, Marta Sitges, Lluis Mont, Ivo Roca-Luque, Till F Althoff

**Affiliations:** Department of Cardiology, Cardiovascular Institute (ICCV), CLÍNIC—Barcelona University Hospital, Carrer Villarroel 170, 08036 Barcelona, Catalonia, Spain; Department of Cardiology, Cardiovascular Institute (ICCV), CLÍNIC—Barcelona University Hospital, Carrer Villarroel 170, 08036 Barcelona, Catalonia, Spain; Department of Cardiology, Cardiovascular Institute (ICCV), CLÍNIC—Barcelona University Hospital, Carrer Villarroel 170, 08036 Barcelona, Catalonia, Spain; Department of Cardiology, Cardiovascular Institute (ICCV), CLÍNIC—Barcelona University Hospital, Carrer Villarroel 170, 08036 Barcelona, Catalonia, Spain; Department of Cardiology, Cardiovascular Institute (ICCV), CLÍNIC—Barcelona University Hospital, Carrer Villarroel 170, 08036 Barcelona, Catalonia, Spain; Institut d’Investigacions Biomèdiques August Pi i Sunyer (IDIBAPS), Department of Arrhythmia Research, C/del Rosselló, 149, 08036 Barcelona, Catalonia, Spain; Department of Cardiology, Cardiovascular Institute (ICCV), CLÍNIC—Barcelona University Hospital, Carrer Villarroel 170, 08036 Barcelona, Catalonia, Spain; Institut d’Investigacions Biomèdiques August Pi i Sunyer (IDIBAPS), Department of Arrhythmia Research, C/del Rosselló, 149, 08036 Barcelona, Catalonia, Spain; Department of Cardiology, Cardiovascular Institute (ICCV), CLÍNIC—Barcelona University Hospital, Carrer Villarroel 170, 08036 Barcelona, Catalonia, Spain; Institut d’Investigacions Biomèdiques August Pi i Sunyer (IDIBAPS), Department of Arrhythmia Research, C/del Rosselló, 149, 08036 Barcelona, Catalonia, Spain; Department of Cardiology, Cardiovascular Institute (ICCV), CLÍNIC—Barcelona University Hospital, Carrer Villarroel 170, 08036 Barcelona, Catalonia, Spain; Institut d’Investigacions Biomèdiques August Pi i Sunyer (IDIBAPS), Department of Arrhythmia Research, C/del Rosselló, 149, 08036 Barcelona, Catalonia, Spain; Department of Cardiology, Cardiovascular Institute (ICCV), CLÍNIC—Barcelona University Hospital, Carrer Villarroel 170, 08036 Barcelona, Catalonia, Spain; Institut d’Investigacions Biomèdiques August Pi i Sunyer (IDIBAPS), Department of Arrhythmia Research, C/del Rosselló, 149, 08036 Barcelona, Catalonia, Spain; Department of Cardiology, Cardiovascular Institute (ICCV), CLÍNIC—Barcelona University Hospital, Carrer Villarroel 170, 08036 Barcelona, Catalonia, Spain; Institut d’Investigacions Biomèdiques August Pi i Sunyer (IDIBAPS), Department of Arrhythmia Research, C/del Rosselló, 149, 08036 Barcelona, Catalonia, Spain; Department of Cardiology, Cardiovascular Institute (ICCV), CLÍNIC—Barcelona University Hospital, Carrer Villarroel 170, 08036 Barcelona, Catalonia, Spain; Institut d’Investigacions Biomèdiques August Pi i Sunyer (IDIBAPS), Department of Arrhythmia Research, C/del Rosselló, 149, 08036 Barcelona, Catalonia, Spain; Department of Cardiology, Cardiovascular Institute (ICCV), CLÍNIC—Barcelona University Hospital, Carrer Villarroel 170, 08036 Barcelona, Catalonia, Spain; Institut d’Investigacions Biomèdiques August Pi i Sunyer (IDIBAPS), Department of Arrhythmia Research, C/del Rosselló, 149, 08036 Barcelona, Catalonia, Spain; Centro de Investigación Biomédica en Red, Cardiovascular Diseases (CIBERCV), Av. Monforte de Lemos, 3-5, 28029 Madrid, Spain; Department of Cardiology, Cardiovascular Institute (ICCV), CLÍNIC—Barcelona University Hospital, Carrer Villarroel 170, 08036 Barcelona, Catalonia, Spain; Institut d’Investigacions Biomèdiques August Pi i Sunyer (IDIBAPS), Department of Arrhythmia Research, C/del Rosselló, 149, 08036 Barcelona, Catalonia, Spain; Centro de Investigación Biomédica en Red, Cardiovascular Diseases (CIBERCV), Av. Monforte de Lemos, 3-5, 28029 Madrid, Spain; Department of Cardiology, Cardiovascular Institute (ICCV), CLÍNIC—Barcelona University Hospital, Carrer Villarroel 170, 08036 Barcelona, Catalonia, Spain; Institut d’Investigacions Biomèdiques August Pi i Sunyer (IDIBAPS), Department of Arrhythmia Research, C/del Rosselló, 149, 08036 Barcelona, Catalonia, Spain; Centro de Investigación Biomédica en Red, Cardiovascular Diseases (CIBERCV), Av. Monforte de Lemos, 3-5, 28029 Madrid, Spain; Department of Cardiology, Cardiovascular Institute (ICCV), CLÍNIC—Barcelona University Hospital, Carrer Villarroel 170, 08036 Barcelona, Catalonia, Spain; Institut d’Investigacions Biomèdiques August Pi i Sunyer (IDIBAPS), Department of Arrhythmia Research, C/del Rosselló, 149, 08036 Barcelona, Catalonia, Spain; Centro de Investigación Biomédica en Red, Cardiovascular Diseases (CIBERCV), Av. Monforte de Lemos, 3-5, 28029 Madrid, Spain; Department of Cardiology, Cardiovascular Institute (ICCV), CLÍNIC—Barcelona University Hospital, Carrer Villarroel 170, 08036 Barcelona, Catalonia, Spain; Institut d’Investigacions Biomèdiques August Pi i Sunyer (IDIBAPS), Department of Arrhythmia Research, C/del Rosselló, 149, 08036 Barcelona, Catalonia, Spain; Department of Cardiology, Cardiovascular Institute (ICCV), CLÍNIC—Barcelona University Hospital, Carrer Villarroel 170, 08036 Barcelona, Catalonia, Spain; Institut d’Investigacions Biomèdiques August Pi i Sunyer (IDIBAPS), Department of Arrhythmia Research, C/del Rosselló, 149, 08036 Barcelona, Catalonia, Spain; Centro de Investigación Biomédica en Red, Cardiovascular Diseases (CIBERCV), Av. Monforte de Lemos, 3-5, 28029 Madrid, Spain; Department of Cardiology, Cardiovascular Institute (ICCV), CLÍNIC—Barcelona University Hospital, Carrer Villarroel 170, 08036 Barcelona, Catalonia, Spain; Institut d’Investigacions Biomèdiques August Pi i Sunyer (IDIBAPS), Department of Arrhythmia Research, C/del Rosselló, 149, 08036 Barcelona, Catalonia, Spain; Centro de Investigación Biomédica en Red, Cardiovascular Diseases (CIBERCV), Av. Monforte de Lemos, 3-5, 28029 Madrid, Spain; Department of Cardiology, Cardiovascular Institute (ICCV), CLÍNIC—Barcelona University Hospital, Carrer Villarroel 170, 08036 Barcelona, Catalonia, Spain; Institut d’Investigacions Biomèdiques August Pi i Sunyer (IDIBAPS), Department of Arrhythmia Research, C/del Rosselló, 149, 08036 Barcelona, Catalonia, Spain; Centro de Investigación Biomédica en Red, Cardiovascular Diseases (CIBERCV), Av. Monforte de Lemos, 3-5, 28029 Madrid, Spain; Department of Cardiology, Cardiovascular Institute (ICCV), CLÍNIC—Barcelona University Hospital, Carrer Villarroel 170, 08036 Barcelona, Catalonia, Spain; Institut d’Investigacions Biomèdiques August Pi i Sunyer (IDIBAPS), Department of Arrhythmia Research, C/del Rosselló, 149, 08036 Barcelona, Catalonia, Spain

**Keywords:** Atrial fibrillation, Catheter ablation, Same-day discharge, Ambulatory, Coordinator, Advanced practice nurse, Nurse-led care, Ambulatory cardiac intervention nurse

## Abstract

**Aims:**

Same-day discharge (SDD) after atrial fibrillation (AF) ablation is an effective means to spare healthcare resources. However, safety remains a concern, and besides structural adaptations, SDD requires more efficient logistics and coordination. Therefore, in this study, we implement a streamlined, nurse-coordinated SDD programme following a standardized protocol.

**Methods and results:**

As a dedicated SDD coordinator, a nurse specialized in ambulatory cardiac interventions was in charge of the full SDD protocol, including eligibility, patient flow, in-hospital logistics, patient education, and discharge as well as early post-discharge follow-up by smartphone-based virtual visits. Patients planned for AF ablation were considered eligible if they had a left ventricular ejection fraction (LVEF) ≥35%, with basic support at home and accessibility of the hospital within 60 min also forming a part of the eligibility criteria. A total of 420 consecutive patients were screened by the SDD coordinator, of whom 331 were eligible for SDD. The reasons for exclusion were living remotely (29, 6.9%), lack of support at home (19, 4.5%), or LVEF <35% (17, 4.0%). Of the eligible patients, 300 (91%) were successfully discharged the same day. There were no major post-SDD complications. Rates of unplanned medical attention (19, 6.3%) and 30-day readmission (5, 1.6%) were extremely low and driven by femoral access–site complications. These were significantly reduced upon the introduction of compulsory ultrasound-guided punctures after the initial 150 SDD patients (*P* = 0.0145). Standardized SDD coordination resulted in efficient workflows and reduced the total workload of the medical staff.

**Conclusion:**

Same-day discharge after AF ablation following a nurse-coordinated standardized protocol is safe and efficient. The concept of ambulatory cardiac intervention nurses functioning as dedicated coordinators may be key in the future transition of hospitals to SDD. Ultrasound-guided femoral puncture virtually eliminated relevant femoral access–site complications in our cohort and should therefore be a prerequisite for SDD.

## Graphical Abstract

**Graphical Abstract:**
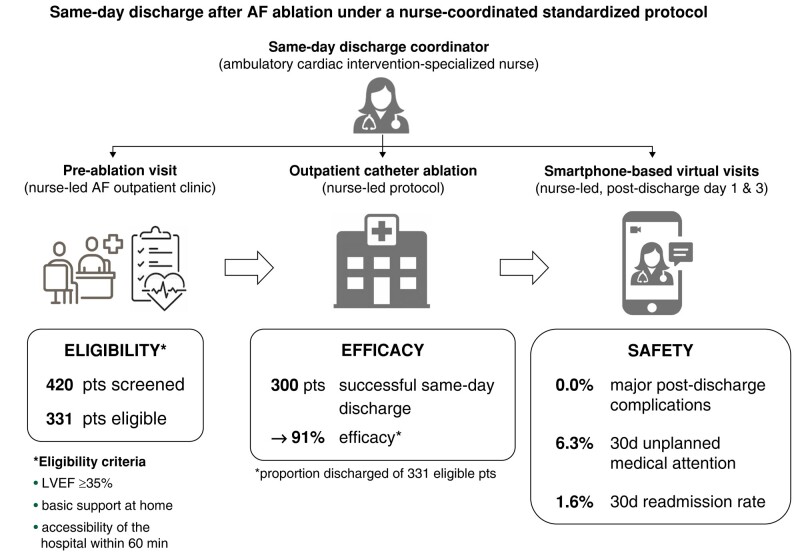

**What’s new?**

- Same-day discharge (SDD) fully coordinated by an ambulatory cardiac intervention–specialized nurse following a standardized protocol is feasible, effective, and safe.
- Implementation of a dedicated coordinator with a standardized protocol streamlines the SDD workflow and in-hospital logistics.
- Ultrasound-guided femoral puncture virtually eliminated femoral access–site complications triggering unplanned medical attention and readmissions and should therefore be a prerequisite for SDD.

## Introduction

Catheter ablation in terms of pulmonary vein isolation (PVI) constitutes the most effective therapy for atrial fibrillation (AF) to date.^1,2^ In fact, PVI was consistently shown in randomized trials not only to reduce the risk of arrhythmia recurrence and AF burden, but also to prevent disease progression from paroxysmal to persistent AF.^3,4^

However, PVI is a complex, resource-intensive procedure, and it constitutes an increasing challenge for healthcare systems worldwide to accommodate the increasing demand for AF ablation, due to limitations in healthcare budget and personnel as well as structural resources. The estimated exponential rise in AF prevalence and increased age-adjusted incidence will further aggravate the mismatch between supply and demand.^2^ Same-day discharge (SDD) is an effective way to mitigate this imbalance by reducing the utilization of healthcare resources, including personnel and hospital beds.^5,6^ However, in the context of abbreviated post-ablation monitoring, safety remains a concern.^7–10^ Moreover, besides structural adaptations, SDD requires more efficient logistics and coordination.^11–13^ Against this background, we evaluated the safety and efficiency of a streamlined SDD programme for AF ablation fully coordinated by a nurse specialized in ambulatory cardiac interventions by following a standardized protocol.

## Methods

### Study design

This was a prospective register-based observational study that included patients undergoing AF ablation between April 2021 and September 2023. Consecutive patients were included based on prespecified eligibility criteria (see below). The study was approved by the ethics committee of our institution, and written informed consent was obtained from each patient.

### Patient eligibility for same-day discharge

Patients were screened during the pre-ablation visit at the nurse-led AF outpatient clinic (1–2 weeks prior to ablation) based on the predefined SDD eligibility criteria and scheduled accordingly—either for the SDD AF ablation programme or for an inpatient AF ablation. The final assignment was authorized by a senior electrophysiologist.

The eligibility criteria were as follows:

- Patients with planned AF ablation (first-time or repeat ablation)
- Proximity of patient residence (accessibility of the hospital within 60 min)
- Support person (e.g. family member) to accompany the patient and provide basic assistance back home
- Left ventricular ejection fraction ≥ 35%

Patients could also be excluded at the operator’s discretion or based on patient preference.

### Same-day discharge coordinator position

At our centre, we established an ambulatory service that was closely interlinked with our nurse-led AF outpatient clinic. For the implementation of a streamlined AF ablation SDD programme, we developed a standardized protocol and created the position of a dedicated SDD coordinator that was staffed by a nurse specialized in cardiovascular ambulatory interventions. These specifically trained nurses attended to all ambulatory cardiovascular procedures, including catheter ablations, cardiac implantable electronic device operations, percutaneous coronary interventions, transcatheter valve replacements or repairs, and other structural cardiac interventions.

**Figure 1:**
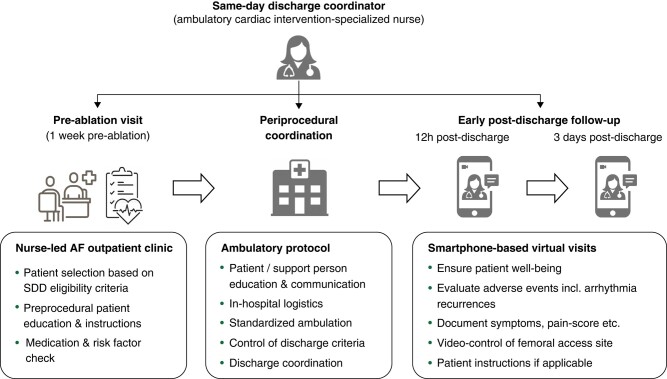
Same-day discharge coordinator work flow. AF, atrial fibrillation; SDD, same-day discharge.

As SDD coordinator, an ambulatory cardiac intervention nurse was in charge of the full SDD protocol, including patient selection (in collaboration with the nurse-led AF outpatient clinic), patient flow, in-hospital logistics, patient and family education, patient discharge, and short-term follow-up (*Figure 1*, see details below).

### Preprocedural preparation

All patients were admitted to a cardiac short-stay unit at a minimum of 1 h before the scheduled ablation. The patients were informed, and written consent was obtained by the operator and the anaesthesiologist, respectively. At this point, the SDD eligibility criteria were re-evaluated by the SDD coordinator.

### Ablation procedure

The procedural methodology has been described previously.^14,15^ In brief, the patients underwent catheter ablation under uninterrupted anticoagulation if treated with vitamin K antagonists. In case of direct oral anticoagulant treatment, a single dose–interrupted anticoagulation regimen was followed. All catheter ablation procedures were performed under general anaesthesia. The ablation approaches and technologies employed were at the operator’s discretion and included conventional or high power-short duration radiofrequency (RF) ablation (Biosense Webster, Abbott, or Boston Scientific), cryoballoon ablation (Medtronic), or pulsed field ablation (PFA, Farapulse).

The use of ultrasound-guided venous access was left to the operator’s discretion in the initial cohort of the first 150 SDD patients, but became compulsory for all subsequent patients. Two to three right femoral venous sheaths were introduced with maximum inner diameters of 8.5 F (RF ablation), 12 F (cryoballoon ablation), or 13 F (PFA). Femoral arterial sheaths were not used.

Transseptal access was accomplished under fluoroscopic guidance; additional guidance by transoesophageal echo was left to the operator’s discretion. Once left atrial access was established, boli of intravenous heparin were administered to maintain an activated clotting time of ≥ 300 s.

After ablation, all sheaths were removed immediately using a ‘figure-of-8’ suture and a compression bandage for haemostasis. No vascular closure devices were employed. Protamine was reserved only for the management of bleeding complications. In all patients, echocardiography was performed before leaving the EP lab to rule out pericardial effusion.

### Post-ablation care

Following ablation, the patients maintained bedrest for at least 4 h and were monitored at the recovery area of the cardiac short-stay unit for at least 6 h: After 4 h, an echocardiographic control was performed to rule out a pericardial effusion before resuming oral anticoagulation. After 5 h, the femoral compression and the figure-of-8 suture were removed, followed by a standardized ambulation protocol executed by the SDD coordinator. If haemostasis was not obtained, or ambulation was unsuccessful, bedrest was extended.

### Patient discharge

The patient and the accompanying family or support person were provided with comprehensive discharge instructions by the SDD coordinator. Among other things, they were specifically instructed how to react and whom to contact in case of complications, including bleeding from the puncture site, recurrent arrhythmias, or chest pain.

Anti-arrhythmic drug therapy was by default maintained unchanged until the 3-month follow-up visit, unless otherwise prescribed by the attending cardiologist. Any changes in medication had to be specifically prescribed by the attending cardiologist.

Prior to discharge, the femoral access site was inspected by the SDD coordinator to rule out a manifest vascular complication.

The criteria listed below were prerequisites for patient discharge. However, the final decision to either admit a patient for overnight monitoring or discharge him/her into the care of his/her family or a support person was left to the discretion of the attending cardiologist.

The discharge criteria were as follows:

- Absence of procedural or post-procedural complications
- Haemodynamic stability, defined as an adequate blood pressure and heart rate, i.e. the absence of relevant hypotension (<90/60 mmHg) or hypertension (>160/100  mmHg) and absence of relevant bradycardia (<50/min) or tachycardia (>100/min)
- Spontaneous micturition
- Tolerance of oral nutritional intake and absence of post-operative nausea and vomiting
- Uncomplicated recovery and successful ambulation
- Absence of femoral access–site bleeding after ambulation without compression and under adequate anticoagulation
- Pre-discharge echocardiography, femoral vascular auscultation, and electrocardiogram (ECG) without findings
- Re-initiation of adequate anticoagulation
- Completion of the ablation procedure before 3:00 PM (logistic reasons—to ensure the minimum of 6 h monitoring at the cardiac short-stay unit, obviating the need for a night shift after 10:00 PM)

### Study endpoints

#### Efficacy

The primary efficacy endpoint was defined as successful SDD after AF ablation.

#### Safety

The primary safety endpoints were as follows:

1. Unplanned medical attention within 30 days after discharge
2. Hospital readmission of any cause within 30 days after discharge
3. Composite safety outcome, including the following post-discharge complications:
  - Death from any cause
  - Stroke, transient ischemic attack (TIA), or systemic embolism
  - Pericardial effusion or cardiac tamponade
  - Moderate or major bleeding [Type 3 or 5 according to the Bleeding Academic Research Consortium (BARC) Definition for Bleeding]^16^

### Follow-up

- Days 1 and 3: smartphone-based virtual visit (SDD coordinator): As part of the SDD protocol, the SDD coordinator set up calls with the patients on post-discharge Days 1 and 3 to ensure their well-being and the good aspect of the femoral access site (smartphone video control). Moreover, based on a standardized protocol, during those visits, the ambulatory cardiac intervention nurse specifically interrogated patients regarding symptoms suggestive of arrhythmia recurrence or procedure-related complications (palpitations, chest pain, dyspnoea, focal neurological deficits, fever, dysphagia, haematemesis, melena, drowsiness, malaise, or right lower extremity pain/discomfort). Pain was monitored based on the numerical rating scale, and analgesic medication was adapted if applicable. Furthermore, adherence to post-discharge instructions and correct medication intake was verified.

The subsequent follow-up was specified by the general AF-ablation protocol of our centre and was not specific for SDD:

- 14 days: in-person visit (nurse-led AF outpatient clinic)
- 3 months: in-person visit (electrophysiologist), ECG, 24 h Holter ECG
- 6 and 12 months: virtual visit (nurse-led AF outpatient clinic), ECG, and 24 h Holter ECG

### Statistical analysis

Statistical analyses were performed using SPSS Statistics 28 (IBM, NY, USA). Descriptive statistics are reported as frequency (percentage) for categorical variables, mean ± standard deviation for normally distributed continuous variables, and median with interquartile range (1st and 3rd quartiles) for non-normally distributed continuous variables. Normal distribution was tested by using the Kolmogorov–Smirnov test. The impact of covariates on unplanned hospital admission or post-discharge medical attention was analysed using unadjusted and adjusted logistic regression models. Variables reaching statistical significance or borderline significance (*P* < 0.10) in the unadjusted regression were included in a multivariate model adjusting for possible confounders through forward stepwise logistic regression. Comparisons of subgroups regarding dichotomous variables were performed using Fisher’s exact test. A two-tailed *P*-value <0.05 was considered significant.

## Results

### Patient characteristics

**Figure 2:**
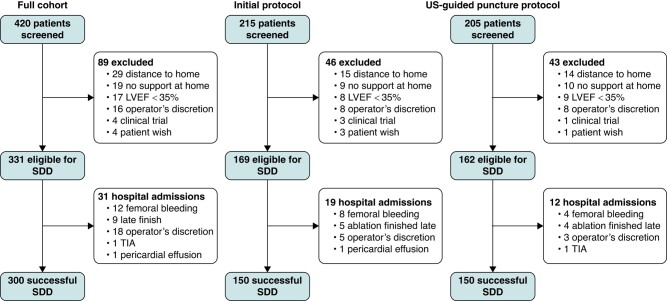
Patient screening and SDD flow chart. LVEF, left ventricular ejection fraction; SDD, same-day discharge.

Between April 2021 and September 2023, 420 patients scheduled for AF ablation were screened for SDD eligibility by the SDD coordinator (96% of all AF ablation patients at our centre). Of those, 331 were included based on the prespecified criteria. The baseline patient characteristics are presented in *Table 1*. On average, the included patients were 61 years old, 27% of them female; the majority had paroxysmal AF (63%). The most frequent reasons for exclusion from SDD were remote patient homes and a lack of basic assistance at home (*Figure 2*). Of note, there were no significant differences in patient characteristics between eligible patients with successful SDD and those who were not discharged the same day.

**Table 1:** Patient characteristics

	Eligible for SDD *n* = 331	SDD realized *n* = 300	SDD not realized *n* = 31	*P*-value
Age	62 (54–67)	62 (54–67)	65 (53–70)	0.45
Female gender	90 (27)	80 (27)	10 (32)	0.54
AF type				
Paroxysmal AF	211 (64)	192 (64)	19 (61)	0.50
Persistent AF	120 (36)	108 (36)	12 (39)	
Hypertension	183 (55)	167 (56)	16 (52)	0.63
Diabetes mellitus	32 (10)	28 (9)	4 (13)	0.66
BMI	27 (24–30)	27 (24–31)	27 (24–30)	0.48
CHA_2_DS_2_-VASc score	1 (1–2)	1 (1–2)	2 (1–3)	0.44
LA diameter, mm	41 (37–46)	41 (37–46)	41 (35–46)	0.23
LVEF, %	60 (55–60)	60 (55–60)	57 (55–60)	0.17
Sleep apnoea	37 (11)	33 (11)	4 (13)	0.50
Previous stroke	17 (5)	15 (5)	2 (6)	0.36

All values are *n* (%) or median with interquartile range (1st and 3rd quartiles). There were no significant differences between cohorts.

AF, atrial fibrillation; BMI, body mass index; LA, left atrial; LVEF, left ventricular ejection fraction; VKA, vitamin K antagonist.

### Same-day discharge efficacy

Of the 331 patients considered for SDD, 300 (91%) were successfully discharged on the day of the ablation procedure. The majority of unplanned overnight hospital admissions were due to minor femoral access–site complications (12, 3.6%) or logistic reasons (late completion of the ablation procedure, 12 patients, 2.7%; see *Figure 2*). Logistic regression analyses, including patient (*Table 1*) and procedural characteristics (*Table 2*) as covariates, revealed the CHA_2_DS_2_-VASc score as an independent predictor of unplanned overnight hospital admission [odds ratio (OR) 1.34, confidence interval (CI) 1.01–1.78, *P* = 0.045; *Table 3*].

**Table 2:** Procedural characteristics

	Eligible for SDD (*n* = 331)	SDD realized (*n* = 300)	SDD not realized (*n* = 31)
Ablation technique			
RF (index-guided)	241 (73)	216 (72)	25 (81)
Cryoballoon	54 (16)	49 (16)	5 (16)
PFA (Farapulse)	36 (11)	35 (12)	1 (3)
Redo ablation	79 (24)	73 (24)	6 (19)
PVI	331 (100)	300 (100)	31 (100)
Additional LA ablation	70 (21)	62 (21)	8 (26)
Peri-procedural anticoagulation			
DOAC	298 (90)	271 (90)	27 (87)
VKA	33 (10)	29 (10)	4 (13)

All values are *n* (%) or median with interquartile range (1st and 3rd quartiles). There were no significant differences between cohorts.

RF, radiofrequency; PFA, pulsed field ablation; PVI, pulmonary vein isolation; LA, left atrial; DOAC, direct oral anticoagulant; SDD, same-day discharge; VKA, vitamin K antagonist.

**Table 3:** Logistic regression

	Unadjusted		
Predictor	Odds ratio	95% CI	*P*-value
Age	1.02	0.98–1.06	0.347
Female gender	1.27	0.59–1.27	0.543
Persistent AF	0.85	0.61–1.19	0.349
Hypertension	0.84	0.42–1.70	0.631
Diabetes mellitus	1.29	0.42–3.96	0.656
CHA_2_DS_2_-VASc	**1**.**34**	**1.01**–**1.78**	**0**.**045**
LA diameter	0.99	0.93–1.05	0.713
LVEF	1.00	0.97–1.02	0.780
Sleep apnoea	0.87	0.37–2.05	0.744
Previous stroke	1.82	0.49–6.72	0.371
Redo ablation	0.78	0.32–1.86	0.569
Additional LA ablation	1.50	0.54–4.16	0.436
Anticoagulation with VKA	1.41	0.48–4.13	0.533

Odds ratios (not considering patients admitted for logistic reasons), 95% CIs, and probability values for predictors of hospital admission in patients planned for SDD. Significant predictors are highlighted by bold letters Unadjusted: unadjusted logistic regression.

AF, atrial fibrillation; LA, left atrial; LVEF, left ventricular ejection fraction; SDD, same-day discharge; VKA, vitamin K antagonist.

### Same-day discharge safety

**Figure 3:**
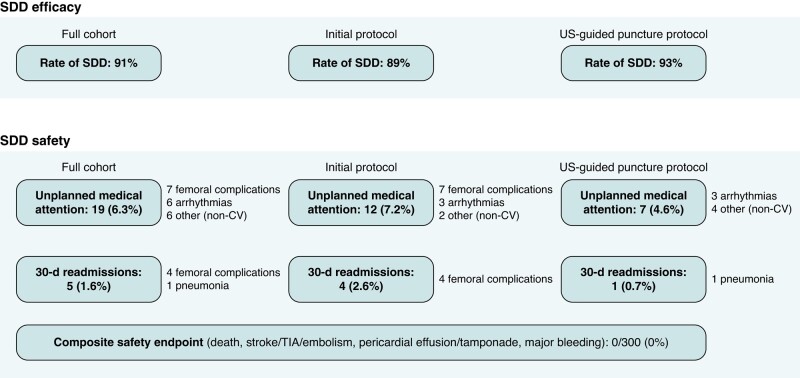
Same-day discharge efficacy and safety. SDD, same-day discharge.

There were no post-discharge complications as defined by the composite safety endpoint [all-cause death; stroke, TIA, or systemic embolism; pericardial effusion or cardiac tamponade; moderate or major bleeding (BARC 3–5)] in any of the 300 SDD patients (*Figure 3*). However, 19 patients (6.3%) sought unplanned medical attention within 30 days of discharge. The 30-day hospital readmission rate was 1.6% (five patients). According to logistic regression analyses adjusting for possible confounders, female sex was the only independent predictor of unplanned medical attention within 30 days of discharge (OR 8.17, CI 2.10–31.71, *P* = 0.002; *Table 4*).

**Table 4:** Logistic regression

	Unadjusted			Adjusted		
Predictor	Odds ratio	95% CI	*P*-value	Odds ratio	95% CI	*P*-value
Age	1.04	0.99–1.15	0.109			
Female gender	**8**.**17**	**2.10**–**31.71**	**0**.**002**	**12.38**	**2.43–62.94**	**0.002**
Persistent AF	1.05	0.73–1.50	0.809			
Hypertension	0.95	0.28–3.20	0.936			
Diabetes mellitus	—	—	—			
CHA_2_DS_2_-VASc score	**1**.**50**	**0.93**–**2.42**	**0**.**070**			
LA diameter	1.00	0.89–1.11	0.948			
LVEF	1.09	0.96–1.23	0.179			
Sleep apnoea	0.58	0.09–3.68	0.564			
Previous stroke	2.02	0.24–17.07	0.520			
Redo ablation	0.17	0.30–4.55	0.819			
Additional LA ablation	**3**.**54**	**0.89**–**14.09**	**0**.**073**			
Anticoagulation with VKA	0.27	0.02–4.08	0.344			

Odds ratios, 95% CIs, and probability values for predictors of unplanned medical attention. Unadjusted: unadjusted logistic regression. Adjusted: variables reaching statistical significance or borderline significance (*P* < 0.10, bold font) were included in a multivariable model adjusting for possible confounders through forward stepwise logistic regression.

AF, atrial fibrillation; LA, left atrial; LVEF, left ventricular ejection fraction; SDD, same-day discharge; VKA, vitamin K antagonist.

Of note, both 30-day unplanned medical attention and hospital readmissions were largely driven by femoral access–site complications. Based on an interim analysis after 150 SDD patients (initial protocol), which identified femoral access–site complications as the main reason for overnight hospital admissions and the dominant driver of unplanned post-discharge medical attention, the SDD protocol was changed and compulsory ultrasound guidance of femoral puncture introduced (US-guided puncture protocol). Indeed, unplanned medical attention due to femoral complications was significantly reduced upon the introduction of mandatory ultrasound-guided puncture: seven patients (4.6%) of the initial cohort, but no patient (0%) of the subsequent ultrasound-guided puncture cohort, had femoral access–site complications triggering unplanned medical attention (*P* = 0.0145).

Arrhythmias were the second most common reason for unplanned medical attention (6, 2%).

## Discussion

In this study, we report our experience with a newly implemented SDD programme for AF ablation. Our data demonstrate that SDD after AF ablation based on a standardized protocol and fully coordinated by an advanced practice nurse within the framework of a nurse-led AF clinic is effective, efficient, and safe.

### Same-day discharge efficacy

Of the 420 patients screened by the SDD coordinator, 79% were considered eligible for SDD, with logistic aspects such as remote patient homes and a lack of basic assistance at home being the most frequent reasons for exclusion, indicating a representative, non-selective population. The vast majority (91%) of the eligible patients were successfully discharged on the day of the ablation—an efficacy rate similar to those reported recently by Deyell *et al*. and Jimenez-Candil *et al*.^6,8^ Of note, bleeding at the femoral access site was the most frequent cause of unplanned overnight hospital admission, followed by logistic reasons (late completion of the ablation procedure). These were also the most common reasons for hospital admission in recent studies by Deyell *et al*.^8,9^ Regression analyses found the CHA_2_DS_2_-VASc score to be an independent predictor of unplanned hospital admission—an aspect that may be considered in the planning of SDD programmes. While the CHA_2_DS_2_-VASc score has never been specifically analysed as a predictor in this context, previous studies consistently reported an association of individual CHA_2_DS_2_-VASc components with unsuccessful SDD.^8,9,17^

### Same-day discharge safety

Importantly, there were no primary safety events in terms of severe post-discharge complications, and 30-day rates of unplanned medical attention or hospital readmission after discharge were extremely low. In fact, the observed rates of unplanned medical attention or readmission within 30 days after SDD were even lower than previously reported for similar cohorts.^6,8,9,18^ This may reflect the positive impact of the standardized ambulation and discharge protocol, including a comprehensive education of patients and support persons, as well as the close follow-up with virtual visits on post-discharge Days 1 and 3—all provided by the SDD coordinator. In addition, all patients were scheduled for inpatient visits at 15 days (nurse-led AF outpatient clinic) and 3 months (electrophysiologist), according to the general AF ablation protocol of our centre (irrespective of SDD), which may also have helped to avoid unplanned medical visits.

### Ultrasound guidance for femoral puncture

Both 30-day unplanned medical attention and readmissions were largely driven by femoral access–site complications. It is particularly noteworthy that femoral access–site complications triggering medical contact were virtually eliminated by the implementation of compulsory ultrasound-guided femoral puncture after an interim analysis of the first 150 SDD patients. This finding is in line with that of previous reports on systematic ultrasound-guided femoral venous puncture and implies that ultrasound guidance is advisable, particularly in the context of SDD.^19–22^

### Early atrial fibrillation recurrence

Another important reason for unplanned medical attention was early arrhythmia recurrence. Although this type of complication is not specific to SDD, it indicates the clinical significance of recurrent AF during the early post-ablation period, which is usually considered the blanking period.^23,24^

### Female sex predicts unplanned medical attention after same-day discharge

Interestingly, female sex was the only independent predictor of unplanned medical attention within 30 days after SDD. Of note, a recent study by Sahashi *et al*.^17^ similarly found female sex to be an independent predictor of hospital readmission. However, as vascular access-site complications and arrhythmia recurrences did not differ significantly between women and men, we could only speculate about potential explanations, and a putative causal relationship remains to be elucidated.

### Same-day discharge efficiency and challenges

Same-day discharge is an effective way to reduce the utilization of healthcare resources, including personnel and hospital beds, and may allow for the accommodation of increasing numbers of AF ablations in the future. This efficiency and cost-effectiveness of SDD after AF ablation have been demonstrated previously.^5,6,9,10^ However, the overall saving of time, personnel, and hospital beds does not come without a cost. Besides structural adaptations, SDD also requires more efficient in-hospital logistics and coordination as well as organizational efforts. Further, SDD inevitably shifts responsibilities to the patient and his family or support person. This requires intensified communication and collaboration with the patient and his company, including discharge education and specific instructions.

In the traditional inpatient setting, no personnel are specifically designated and budgeted for these challenges, and the responsibilities at this interface of ambulatory and in-hospital care are often not well defined among nurses and physicians. Thus, new SDD-specific tasks will have to be accomplished by the existing medical staff, further increasing their already dense workload.

### General applicability of the results

Our intention in this study was to investigate an unselected all-comer AF ablation population, and indeed, the screening rate was quite complete: 96% of all patients undergoing AF ablation at our centre were screened for SDD eligibility and included in the analysis. The screening rate was even higher in the second half of the study (initial cohort: 94%; subsequent, US-guided puncture cohort: 99%; *P* = 0.013). This might be explained by the fact that the nurse-led coordination with its workflow including patient screening still had to be established in the daily routine. Importantly, the rate of successful SDD was numerically even higher in the subsequent cohort, indicating that, despite a slightly less complete screening, there was no selection bias in the initial cohort.

### Concept of ambulatory cardiac intervention–specialized nurses

It is increasingly acknowledged that SDD has great potential to mitigate the challenges that hospitals are facing in the context of invasive cardiovascular medicine, and the resource constraints and immense pressure on the healthcare system are likely to lead to a speedy and broad implementation of SDD. However, this is an emerging field, and SDD will require considerable adaptations and novel concepts with newly defined responsibilities, positions, and perhaps even professions.

Against this background, to ensure an efficient, well-coordinated SDD workflow and to unburden the existing medical staff, we developed the concept of a nurse-led ambulatory cardiac intervention service that was closely interlinked with our nurse-led AF outpatient clinic, and we created the position of a dedicated SDD coordinator. As mentioned above, the SDD coordinator was responsible for implementing the full SDD protocol, from patient selection to post-ablation follow-up, taking care of all SDD-specific tasks.

Of note, this previously undefined position implies a novel concept of nurses specialized in ambulatory cardiac interventions. This concept requires nurses to have specific training and gain experience in the peri-procedural care of patients undergoing invasive cardiovascular procedures. Ambulatory cardiac interventional nursing may be a highly attractive professional path that provides exciting perspectives to nurses in cardiovascular medicine. While there is little doubt that in the near future there will be an immense demand for specialized nurses who are trained accordingly, such a concept will need standardized training and education programmes as well as acknowledgement by cardiac and nursing societies, and ultimately perhaps even official legitimation through board certification.

### Same-day discharge coordinator impact

While we cannot provide definite evidence, it was apparent that the implementation of a dedicated SDD coordinator position streamlined the whole SDD protocol and patient pathway. It resulted in a noticeable workload reduction for physicians and nurses alike and significantly improved communication among all relevant parties, thus leading to a great acceptance of the SDD programme. These findings are consistent with those reported for transcatheter aortic valve replacement programmes, where the implementation of specialized nurse coordinators has been shown to render patient pathways more efficient and to reduce overall workload, while improving interaction between patients and medical staff.^25,26^ However, quantitative data in the context of catheter ablation are lacking.^27^ Moreover, although again we cannot provide quantitative data, the feedback clearly indicates that the systematic interaction and patient education provided by the SDD coordinator and its function as a contact person increased patient satisfaction considerably. Finally, a systematic coordination of workflows and patient pathways, as well as standardized education and specific instructions provided by the SDD coordinator, might even have contributed to the compelling safety outcome.

### Limitations

The fact that there is no control group without SDD is a limitation that prohibits definite quantitative comparisons. However, compared with similar cohorts reported previously, the number of post-discharge complications and readmissions was extremely low and convincing *per se*.^6,9^ Women (27%) and particularly elderly patients (21% above the age of 70 years) were relatively underrepresented in the studied population, which has to be considered when interpreting the results and translating them to other populations.

## Conclusions

Same-day discharge after AF ablation following a standardized protocol under the coordination of a specialized nurse is safe and effective. In fact, the position of a dedicated coordinator streamlines the SDD workflow and the whole concept of ambulatory cardiac intervention–specialized nurses may be the key in the future transition of hospitals to SDD, which will minimize the utilization of healthcare resources, including personnel and hospital beds. Importantly, ultrasound-guided femoral puncture virtually eliminated relevant femoral access–site complications in our cohort and should therefore be a prerequisite for SDD.

## Data Availability

The data underlying this article will be shared upon reasonable request to the corresponding author.
